# Effect of Increasing Doses of *γ*-Radiation on Bone Marrow Stromal Cells Grown on Smooth and Rough Titanium Surfaces

**DOI:** 10.1155/2015/359416

**Published:** 2015-07-15

**Authors:** Bo Huang, Mengkai Guang, Jun Ye, Ping Gong, Hua Tang

**Affiliations:** ^1^State Key Laboratory of Oral Diseases, West China Hospital of Stomatology, Sichuan University, Chengdu 610041, China; ^2^Dental Implant Center, West China Hospital of Stomatology, Sichuan University, Chengdu 610041, China

## Abstract

Radiation therapy for oral and maxillofacial tumors could damage bone marrow stromal cells (BMSCs) in jaw, which caused dental implant failure. However, how radiation affects BMSCs on SLA (sandblasted with large-grits, acid-etched) surfaces is still unknown. The aim of this study was to investigate effect of different dose of *γ*-radiation on BMSCs on SLA and PT (polished titanium) surfaces. Rat BMSCs were radiated with 2, 4, and 8 Gy *γ*-radiation and then seeded on both surfaces. Cell adhesion, spreading, and proliferation were tested. The osteogenesis and the adipogenesis ability were examined by Alizarin-Red and Oil-Red staining, respectively. Real-time PCR was performed to detect osteogenic (osteocalcin, OCN; runt-related transcription factor 2, Runx2) and adipogenic (peroxisome proliferator-activated receptor gamma, PPAR*γ*) gene expression at days 7 and 14 postirradiation. Results showed that *γ*-radiation reduced cell proliferation, adhesion, spreading, and osteogenic differentiation. 2 Gy radiation promoted adipogenic differentiation, but it was significantly decreased when dosage reached 4 Gy. In conclusion, results suggest that *γ*-radiation influenced BMSCs behaviors in a dosage-dependent manner except adipogenic differentiation, low dose promoted it, and high dose inhibited it. This effect was influenced by surface characteristics, which may explain the different failure rate of various implants in patients after radiation.

## 1. Introduction

As a clinical therapy to treat oral and maxillofacial tumors, radiation therapy could be an adjuvant access in combination with ablative surgery to prolong the long time survival rate. DNA damage, apoptosis, cell cycle arrest, mutagenesis, and nucleotide excision repair, however, would be induced by radiation and cell injury as a result [1, 2]. Moreover, radiation also could induce the production of various forms of reactive oxygen species (ROS) and inflammatory cytokines [3, 4]. These cytokines, including superoxide radicals, hydrogen peroxide, hydroxyl radicals, and tumor necrosis factor-*α* (TNF-*α*) and interleukin-1 (IL-1), were a consequence of persistent DNA-damage signaling associated with senescence [4]. ROS could cause immature cells and further dividing cells damaged and died rapidly. Individual tissues, located in various areas of radiation, reacted with different sensitivities [5]. In the case of bone, especially the adjacent mandible and maxilla, cellular activity, blood supply, and partial oxygen pressure were decreased, therefore, resulting in radiation bone injury (RBI) or even osteoradionecrosis (ORN). Dental implant is a predictable option to replace missing teeth [6], depending on the quality and quantity of the surrounding bone. The incidence of ORN was 4% to 30% after head and neck radiation therapy, which would account for the low success rate of dental implants in radiated bone [7]. Clinical data showed that the risk of implant failure in radiated bone is two to three times greater than that in nonradiated bone [8]. Animal experiments suggested that radiation could inhibit bone regeneration in a dose-dependent manner, and the capacity of bone to integrate with titanium implant, such as bone-to-implant contact (BIC), histological bone area, and biomechanical removal torque, was compromised [9–11]. Asikainen et al. [12] studied different dose of radiation on dental implant using a dog model and demonstrated that 10% of the implants lost and 40% of the implants appeared in the marginal bone resorption under 50 Gy radiation. Moreover, when the radiation reached 60 Gy, all of the implants lost and the supporting bone tissue was absorbed seriously. Li et al. [10] evaluated the dose-dependent effect of radiation on implant stability and osseointegration in the rabbit model. They found that implant stability quotient (ISQ) and ratio of bone volume to total volume (BV/TV) were significantly lower in radiation group than the nonradiation group. And the rate of bone growth and BIC were significantly lower in 30 Gy radiation group than those in 15 Gy radiation group [10].

The characteristics of various implant surfaces, including the rough surface and smooth surface, had a critical influence on the per-implant bone healing [13]. In general, the rough surface can get higher BIC than the machined surface with the same material [14, 15]. Moreover, microtopography induced bone marrow stromal cells (BMSCs) differentiation toward an osteoblast phenotype faster than the smooth surface. And the interaction of topographic features could improve biological response, such as increased cell proliferation and activities, and higher mRNA and protein expression of osteoblast marker. Alkaline phosphatase activity, an early marker of osteogenic differentiation, increased about 50% and osteocalcin, a later marker of osteoblastic differentiation, increased about 200% on modified SLA titanium surfaces compared to PT surfaces [16]. In addition, properties of superficial morphology also play significant roles in cell adhesion and biomolecular adsorption. Osteoblasts plated on SLA surfaces were stretched over the coarse pores even into deep pores and developed multiple points of attachment that were closely associated with the submicrometer features of the surfaces, but cells were flattened without any preferred orientation on PT surfaces. Except these, cells cultured on SLA surfaces were significant thicker than those cultured on PT surface, and more and larger bone like nodules could be seen on SLA surfaces than PT surfaces [17].

As seed cells, multipotential stem cells (MSC) stored in bone marrow, having the ability to differentiate into bone, cartilage, or adipose tissue, are commonly used in dental implant research [18, 19]. The osteogenic differentiation and adipogenic differentiation of BMSCs are reciprocal [20]; several studies indicated that radiation decreased the former but increased the latter [21]. Additionally, clinical studies showed an inverse relationship between trabecular bone tissue and adipose tissue in bone marrow [22].

These results indicate that radiation therapy and the surface structure of Ti surfaces have an important influence on regulation of cell behaviors. However, how SLA and PT titanium (Ti) surfaces affect adipogenetic differentiation of BMSCs and how BMSCs behaviors on different Ti surfaces influenced by radiation are still unclear. The aim of this study was to investigate the effects of different dose of *γ*-radiation on cell proliferation, differentiation, adhesion, spreading, and gene expression (OCN, Runx2, and PPAR*γ* genes) on SLA surface and PT surface. Furthermore, under the same radiation condition, the effects of SLA and PT surfaces on BMSCs were also evaluated.

## 2. Materials and Methods

### 2.1. Ti Surfaces

The titanium discs (1.0 mm in thickness and 14 mm in diameter) (supplied by National Engineering Research Center of Biomaterials, Sichuan University, China) has been analyzed and tested in previous studies in our group [23]. Briefly, for PT surfaces, discs were grounded with a sequence of 500#, 800#, 1500#, and 3000# silicon carbide papers, and for SLA surfaces, discs were blasted with 300 *μ*m alumina oxide particles at a blasting pressure of 4 MPa and then cleaned ultrasonically and dried. After that, the blasted samples were subjected to a double chemical etching using hydrogen peroxide and hydrochloric acid. Before in vitro tests, all titanium discs were ultrasonically cleaned and sterilized in an autoclave. Mahr Perthometer M1 (Mahr, Germany) was used to test surface properties of Ti plates.

### 2.2. Cell Culture

BMSCs were isolated and cultured from rats (supplied by Sichuan University Animal Center, 100 ± 10 g), followed by the protocol described in previous study [24]. Rats were killed through cervical dislocation, tibiae and femurs were removed under aseptic conditions, and bone marrow cells were flushed out with Modified Eagle Medium (DMEM, HyClone, USA). Then, the cells were explanted in DMEM with 10% of Fetal Bovine Serum (FBS, Gibco, Australia), 100 U/mL of penicillin (HyClone, USA), and 100 mg/mL of streptomycin (HyClone, USA) and incubated at 37°C in an atmosphere having 5% CO_2_. Nonadherent cells were discarded twenty-four hours later.

### 2.3. Radiation

Cells were digested by 0.25% trypsin (HyClone, USA) and resuspended with DMEM containing 2% FBS. Then, single dose of 2, 4, and 8 Gy of gamma radiation was administrated, respectively, at a rate of 0.83 Gy/min using a linear accelerator in the Seventh People's Hospital of Chengdu, China. The source-bottle distance was 80 cm and the field of size was 10 × 10 cm^2^. At the same time, control samples were kept outside at the same temperature as the radiated samples. Then, the cells were seeded on SLA and PT surfaces.

### 2.4. Cell Proliferation Assay

Control and radiated cells were seeded on SLA and PT surfaces (1.5 × 10^4^ cells per well). At days 1, 3, 5, and 7, the proliferation of BMSCs was assessed using Cell Counting Kit (CCK-8, Dojindo, Japan) assay. The optical density (OD) values were measured at 450 nm using a microplate reader (Varioskan Flash, Thermo Fisher Scientific, USA).

### 2.5. Cell Attachment on Different Titanium Surfaces

After the cells were seeded on different titanium surfaces for 8 hours and 24 hours, DAPI staining was performed to evaluate the amounts of attached cells under fluorescent microscope (five visual fields for each sample, Olympus IX71-F22FL, Japan) and Scanning Electron Microscope (SEM, HITACHI S3400+EDX, KEKY 2800, Japan) was used to evaluate the morphological features of BMSCs on both surfaces. Briefly, samples were fixed with 2.5% glutaraldehyde for 2 hours and dehydrated with ethanol, treated with dehydration, coated with gold alloy, and inspected by SEM.

### 2.6. Osteogenic Differentiation, Adipogenic Differentiation, and Alkaline Phosphatase Activity Assay

The effect of radiation and Ti surfaces on cell differentiation is tested. Radiated and control cells were seeded on SLA and PT surfaces in 24-well plates (2 × 10^4^ cells per well). Osteogenic differentiation and adipogenic differentiation were performed as follows: cultures were fixed in 4% paraformaldehyde; then, osteogenic differentiation was stained with 1% Alizarin-Red (Sigma, USA) and adipogenic differentiation was stained with 0.3% Oil-Red-O (Sigma, USA). The images were collected with reverse phase contrast microscope (LEICA ZE4 HD, high definition, Germany) and analyzed using Image Pro Plus6. Quantity of calcium mineral was measured by cetylpyridinium chloride (CPC) [25]. To quantify the adipogenic differentiation potential, triglyceride (TG) amounts in the cells were quantified by serum triglyceride determination kit (Sigma, USA). For alkaline phosphatase (ALP) activity assay, cells were collected on the seventh day, washed twice with cold PBS, and lysed with freezing-thawing and ultrasound pyrolysis for three times. Then, they were measured by ALP activity kit (Nanjing Jiancheng Research Institute, China). Total amount protein was measured by bicinchoninic acid (BCA) protein measurement kit (KeyGen Biotech, China).

### 2.7. Real-Time PCR Assay

The effect of radiation and Ti surfaces on gene expression on BMSCs in different groups was assessed. On the seventh day and the fourteenth day, total RNA was extracted with RNA extraction kit (Bioer Technology, China) following the protocol. Concentration of RNA was measured by spectrophotometer, with OD value (A260/A280) between 1.8 and 2.0 reversed to cDNA using Prime Script TM RT-PCR kit (Takara, Japan). The cDNA products were amplified using Takara Taq (DR001AM, Takara, Japan) for 40 cycles (denaturation for 30 s at 95°C, followed by primer annealing for 5 s at 95°C, and extension for 31 s at 60°C). Each real-time PCR was carried out in quadruplicate and performed using ABIPRISM 7300 real-time PCR system (Applied Biosystems, US). GAPDH was used as an internal control and primers sequence were presented in Table 1.

### 2.8. Statistical Analysis

All data were analyzed with SPSS 21.0, using one-way analysis of variation (ANOVA). If the results of ANOVA are statistically significant, Student-Newman-Keuls test is used to analyze the differences. All data are expressed as mean ± SEM. *P* value < 0.05 was statistically significant. Three independent replicates of each experiment were conducted.

## 3. Result

### 3.1. Characterization of Rat BMSCs and Ti Surface Roughness

**Figure 1:**
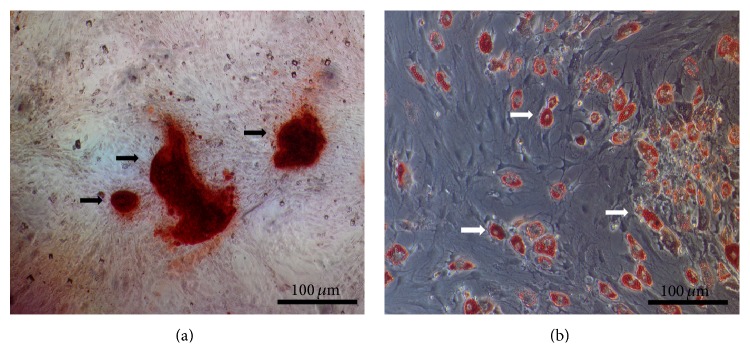
Characterization of rat BMSCs. (a) Alizarin-Red S positive staining of BMSCs was observed after osteogenic inducing for three weeks; black arrows indicate bony nodules. (b) Oil-Red-O-positive staining of BMSCs was observed after adipogenic inducing for ten days; white arrows indicate lipid droplets.

As pluripotent stem cells, the biological property of BMSCs in differentiating to osteoblasts and adipocytes was identified by using Alizarin-Red and Oil-Red-O stain, respectively. Results showed positive stains in Figure 1. The surface roughness (Ra) was 3.243 ± 0.176 for SLA and 0.112 ± 0.015 for PT. And the point heights of irregularities (Rz) of the SLA and PT surfaces were 19.266 ± 1.234 and 1.123 ± 0.169, respectively.

### 3.2. The Effect of Radiation on the Proliferation of BMSCs on Titanium Surface

**Figure 2:**
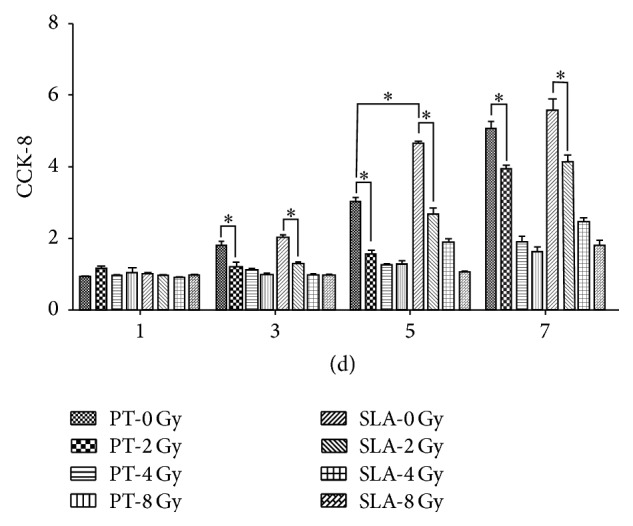
Effects of different dose of radiation on proliferation of BMSCs on PT and SLA surfaces. Radiation inhibited cell proliferation in a dose-dependent manner on both surfaces, and the inhibitor effect was smaller on SLA surface. ^*∗*^ *P* < 0.05. All values expressed as mean ± SD.

As shown in Figure 2, the proliferation of the BMSCs on both PT and SLA surfaces was significantly suppressed when it received radiation (*P* < 0.05), and the higher the dose it received, the more the suppression the results showed. The proliferation almost stopped at the dose of 8 Gy. The proliferation on SLA surface was significantly higher than that on PT surface under 0, 2, and 4 Gy (*P* < 0.05), but with no statistical differences at 8 Gy (*P* > 0.05).

### 3.3. Radiation Reduces Adhesion and Spreading of BMSCs on Different Titanium Surface

**Figure 3:**
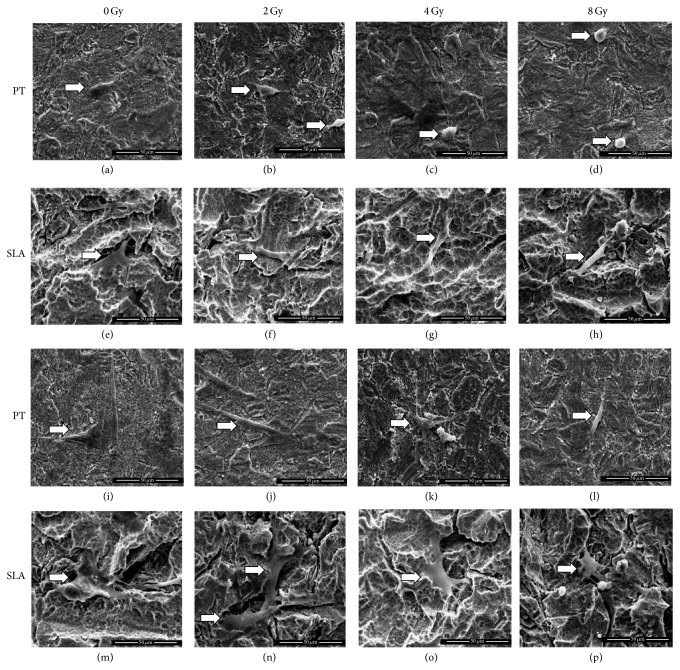
Cell morphological features on different titanium surfaces under different dose of radiation. White arrows indicate the cell on Ti plates. Cell spreading on both surfaces was suppressed under radiation and related to the dosage increased. It is spreading better on SLA surfaces and more pseudopods could be seen on SLA surfaces. (a)–(d): 8 h after radiation on PT surface. (e)–(h): 8 h after radiation on SLA surface. (i)–(l): 24 h after radiation on PT surface. (m)–(p): 24 h after radiation on SLA surface.

**Figure 4:**
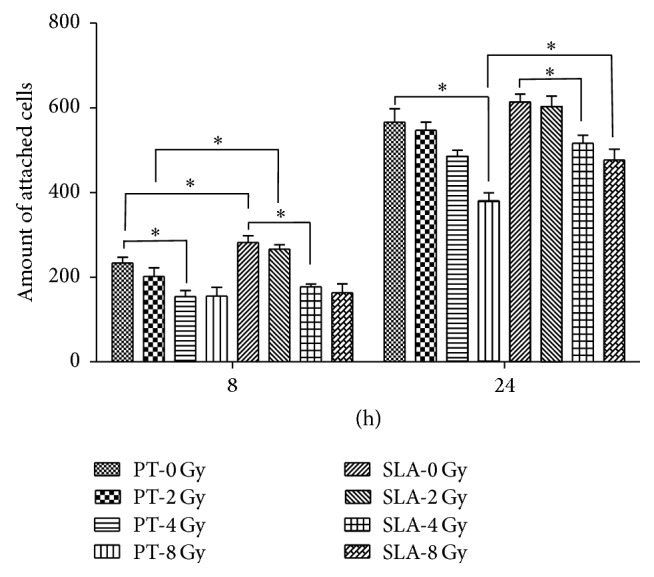
The amount of attached cells on both surfaces. Radiation reduced adhesion of BMSCs on both surfaces in a dose-dependent manner. More adhesion cells were detected on SLA surfaces than on PT surfaces. The difference was smaller on 24 h than 8 h. ^*∗*^ *P* < 0.05.

Figure 3 showed the morphological features of BMSCs on PT and SLA titanium surfaces under different dose of radiation. Overall, cell spreading on both PT and SLA surfaces was reduced after receiving radiation and related to the increased dosage. At 8 h, BMSCs appeared triangle-shaped under 0 or 2 Gy, and ball-shaped on PT surface but elongated-shaped on SLA surface under 4 or 8 Gy. At 24 h, more cellular pseudopods were stretched out under lower dose of radiation. A few pseudopods extended on PT surface, while on SLA surface, more pseudopods were stretched out and fused together. The amounts of BMSCs with different dose of radiation on different titanium surfaces were calculated as Figure 4 shows. At 8 h, attached cells were apparently enhanced on SLA surface comparing to PT surface under 0 and 2 Gy (*P* < 0.05). At 24 h, the differences among groups were smaller.

### 3.4. The Effect of Radiation on Osteogenesis of BMSCs on Different Titanium Surfaces

**Figure 5:**
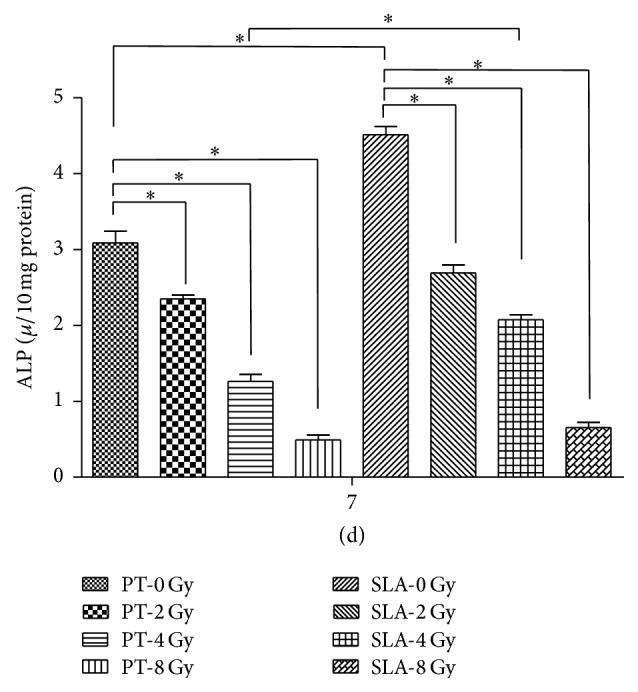
ALP activity was measured at the 7th day. Radiation decreased ALP activity of BMSCs on both surfaces in a dose-dependent manner, and it is higher on SLA surfaces than on PT surfaces. ^*∗*^ *P* < 0.05.

**Figure 6:**
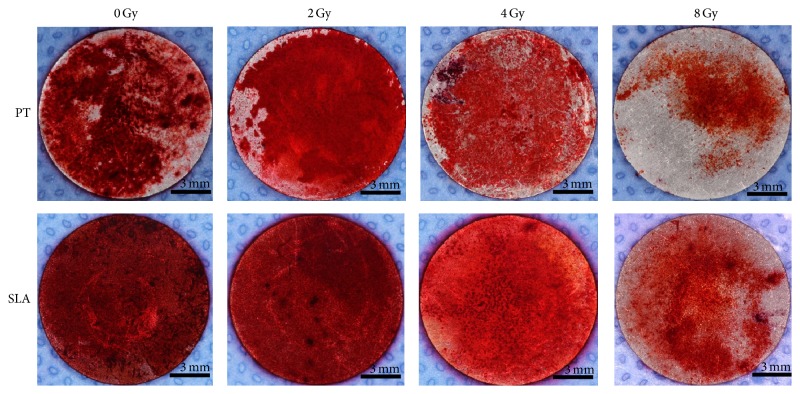
The effect of radiation on osteogenesis of BMSCs on PT and SLA surfaces. Calcium deposition almost covered the whole titanium disc on both PT and SLA surfaces under 0 and 2 Gy. At 8 Gy, the stained area was less than half. And the area was larger on SLA surfaces than on PT surfaces under the same dose of radiation. Red color indicated the positive areas.

**Figure 7:**
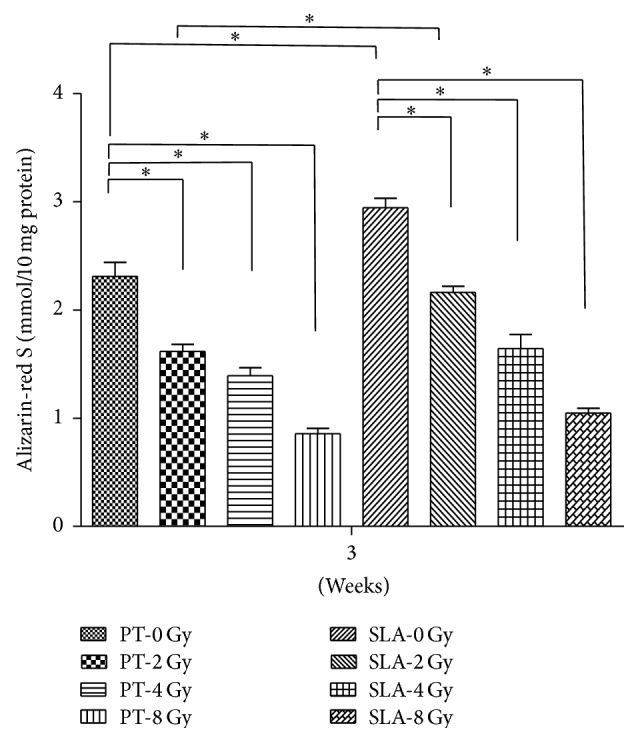
Cetylpyridinium chloride (CPC) was used to measure the quantity of calcium mineralized deposits. The quantity of calcium deposition decreased in a dose-dependent manner, and it is higher on SLA surfaces than PT surfaces. ^*∗*^ *P* < 0.05.

Both calcium deposition and ALP activity were used to measure the osteogenetic differentiation of radiated BMSCs on different titanium surfaces. ALP activity and calcium deposition were reduced in a dose-dependent manner that the higher radiation they received, the less osteogenic ability they exhibited. For ALP activity, the reduction was about 20% at 2 Gy on both surfaces, and about 50% at 4 Gy. At 8 Gy, the reduction was up to almost 80% on both surfaces. Furthermore, ALP activity was higher on SLA surface than that on PT surface with the same dose (*P* < 0.05) except 8 Gy (Figure 5). Alizarin-Red stain showed that calcium deposition almost covered the whole titanium disc on both PT and SLA surfaces under 0 and 2 Gy. At 8 Gy, the stained area was less than half (Figure 6). And the quantity of calcium deposition was shown in Figure 7, which was in accordance with ALP activity.

### 3.5. The Effect of Radiation on Adipogenesis of BMSCs on Different Titanium Surfaces

**Figure 8:**
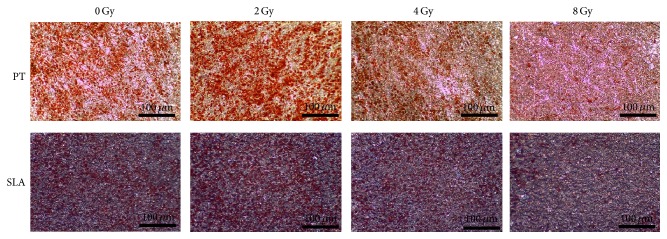
The effect of radiation on adipogenesis of BMSCs on PT and SLA surfaces. Oil-red-O was used to stain lipid droplets of BMSCs after adipogenic induction for 10 days. More positive cells were seen on PT surfaces than on SLA surfaces under the same dose of radiation. However, 2 Gy radiation increased the number of positive cells on both surfaces; 4 Gy and 8 Gy decreased it. Red color indicated the positive cells.

**Figure 9:**
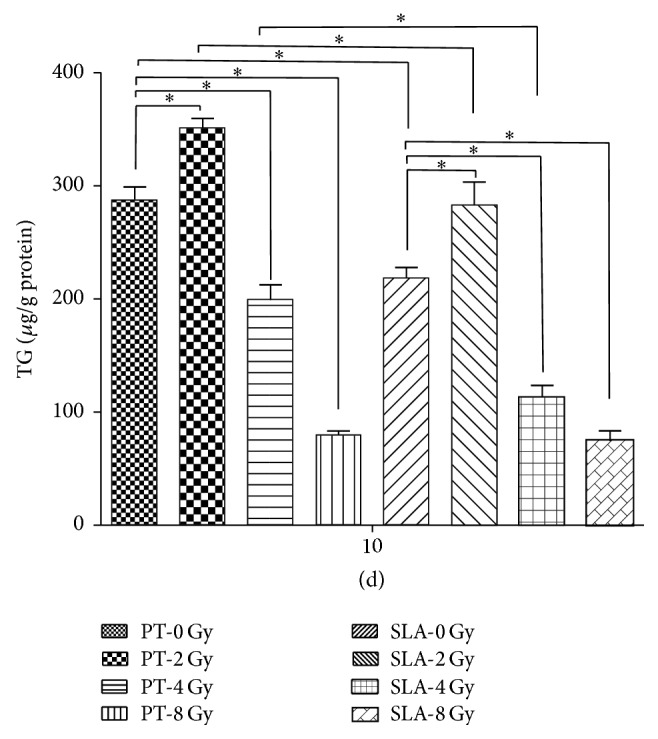
The amount of TG was measured. Under 2 Gy radiation, more TG was detected compared to 0 Gy on both surfaces. However, it significantly decreased at the dose of 4 Gy. There was less TG on SLA surfaces than PT surfaces. ^*∗*^ *P* < 0.05.

At the dose of 2 Gy radiation, more Oil-red-O-positive cells can be seen on both PT surface and SLA surface (Figure 8) compared to 0 Gy, with the amount of triglyceride (TG) increased about 20%. But at the dose of 4 Gy, the amount of TG reduced about 30% on PT surface and 35% on SLA surface, respectively. Moreover, the amount of TG reduced more at dose of 8 Gy. Less TG on SLA surface than that on PT surface at the same dose (*P* < 0.05) was also noted, except 8 Gy (Figure 9).

### 3.6. The Effect of Different Dose of Radiation on Gene Expression on Different Titanium Surfaces

**Figure 10:**
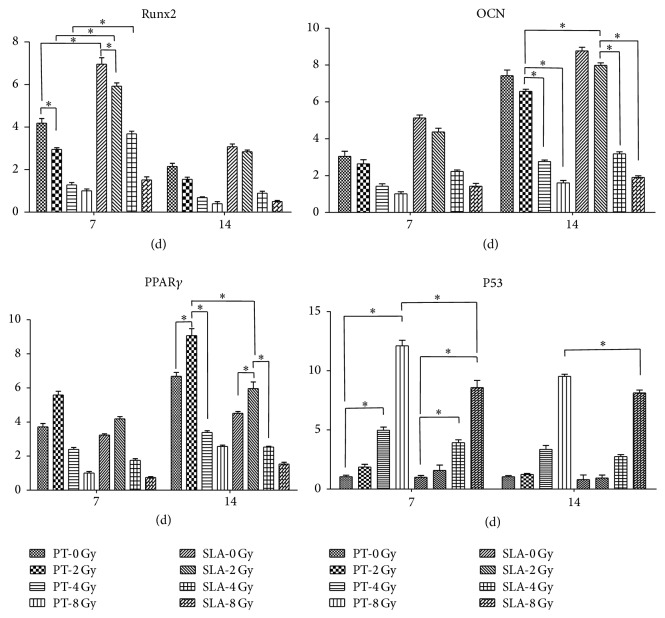
Analysis of mRNAs expression of Runx2, OCN, PPAR*γ*, and P53 under different dose of radiation on PT or SLA surfaces at the 7th and the 14th days. Runx2 was higher at the 7th day than the 14th day under the situation. But OCN was higher at the 14th day. Radiation decreased them in a dose-dependent manner, and they are higher on SLA surfaces than on PT surfaces with the same dose of radiation. PPAR*γ* was higher at the 14th day than the 7th day under the same situation. 2 Gy radiation increased expression of PPAR*γ* on both surfaces. But it significantly decreased with 4 Gy and 8 Gy radiation on both surfaces. And it was higher on PT surfaces than on SLA surfaces with the same dose of radiation. Radiation increased expression of P53 tightly associated with dosage. It was significantly increased under 4 Gy and 8 Gy radiation. P53 was higher at the 7th day than at the 14th day on both surfaces under the same dose of radiation. Compared to PT surfaces, it was lower on SLA surfaces. And the difference was smaller on the 14th day than on the 7th day. ^*∗*^ *P* < 0.05.

Expressions of osteogenic (OCN and Runx2) and adipogenic (PPAR*γ*) gene on PT and SLA surfaces at days 7 and 14 postirradiation are shown in Figure 10. Radiation treatment decreased the expression of OCN and Runx2 relative to GAPDH mRNAs in a dose-dependent manner on both surfaces. The higher the dose they received, the lower the ratio they expressed. The relative Runx2 mRNAs were higher at the 7th day than the 14th day (*P* < 0.05); however, the relative OCN mRNAs were lower at the 7th day (*P* < 0.05). In addition, OCN and Runx2 mRNAs were higher on SLA surface comparing to PT surface.

On the contrary, the relative PPAR*γ* mRNAs were higher at the 14th day than the 7th day on both PT and SLA surfaces (*P* < 0.05). Radiation at dose of 2 Gy increased PPAR*γ* expression on both surfaces further (*P* < 0.05). With radiation at 4 Gy and 8 Gy, nevertheless, the expression of PPAR*γ* mRNAs decreased. On SLA surface, the expression of PPAR*γ* mRNAs was lower than on PT surface under the same dose of radiation.

The apoptosis-related gene P53 was also tested. Radiation increased its expression tightly associated with dosage, and the expression significantly increased when radiation was higher than 4 Gy. It was higher on PT surface than that on SLA surface under the same dose of radiation. At the 7th day, the differences between SLA surface and PT surface were statistical significant under the dose of 8 Gy (*P* < 0.05). At the 14th day, the expression decreased on both surfaces.

## 4. Discussion

Gamma radiation can induce DNA injury via the reactive oxygen species (ROS) directly or indirectly. If normal cells failed to repair the damage, it may result in the cell cycle inhibiting even premature senescence and cell apoptosis [26, 27]. In this study, the proliferation of BMSCs was inhibited by *γ*-radiation in a dose-dependent manner on both PT and SLA surfaces. When the dosage was higher than 4 Gy, the proliferation decreased significantly compared to the dosage of 0 Gy or 2 Gy, which was similar to previous researches [24, 27]. However, some studies reminded us that the dose below 9 Gy or 10 Gy had no noticeable inhibitory effect on cellular proliferative ability [5, 28]. In addition, effect of PT and SLA surfaces on BSMCs exposed to the same dose of radiation was also studied. At the beginning of three days, no significant difference was observed. But at the 5th day, the proliferation rate on SLA surface was higher than that on the PT surface, which is in accordance with previous studies that rougher surfaces were better for cell vitality [29].

It was found that the initial adhesion and spreading activity of cells were the first stage of cell-material interaction, which has great impact on the capacity of cell proliferation and differentiation when contacting with the implant [30]. In vivo and in vitro studies demonstrated that properties of implant surface, such as surface topography, energy, and physical-chemical properties, have significant influence on cell adhesion, morphology, mineralization, and gene expression [31, 32]. Therefore, the morphological features of radiated cells seeded on PT and SLA surfaces after 8 and 24 hours were evaluated. Results showed that *γ*-radiation slowed down the speed of cell spreading and decreased the number of pseudopodia on both surfaces, as well as the amount of attached cells. The higher radiation the cells received, the less pseudopodia they stretched out. And results also suggested that PT surface and SLA surface had different influence on radiated cell-material behaviors, which was consistent with previous studies [5, 17]. Cell adhesion might be affected by radiation through decreasing casein kinase 2 alpha (CK2*α*), which resulted in less Ser1943 phosphorylation of myosin-9 and its redistribution from cytoskeleton to cytoplasm, subsequently, with myosin-10 being reduced and profilin-1 being secreted [33]. Moreover, on account of P53/P21 WAF1 pathway, triggered by IR, involved in cell apoptosis and premature senescence [34, 35], the mRNAs levels of P53 were examined. The difference between 0 Gy and 2 Gy radiation was not significant, when it came to 4 Gy and 8 Gy; however, the level increased about 8 to 10 times. Its expression decreased largely at the 14th day after radiation. And two weeks later after irradiation, cells regained the normal proliferation rate, as described by Li et al. [36].

It was indicated that BMSCs were sensitive to radiation in vivo and in vitro, and IR inhibited osteogenic differentiation of surviving MSCs [24–28, 36]. The activity of ALP and quantity of calcium deposition were used to test the osteogenic potential of BMSCs after radiation. Our research showed that radiation significantly decreased ALP activities as dose increased at the 7th day. Results of Alizarin-Red staining and calcium deposition were similar to ALP activities and were consistent with the previous study [36]. Some studies, nevertheless, demonstrated that cells did not lose their differentiation ability completely even after treatment with high doses [5, 37]. When BMSCs were exposed to the same dose of radiation, SLA surface seemed more conducive to osteogenic differentiation. It maybe accounts for that the osteogenic ability of bone marrow derived cells could be improved on rough surface compared to machined surface, which could synthetize more collagen and express osteogenic gene (COL-I) [38]. Furthermore, as a marker in directing pluripotent mesenchymal cells to preosteoblasts, Runx2 was measured at the genetic level, as well as OCN, a marker in mineralization [39]. Results suggested that radiation affects the osteogenesis gene expression of BMSCs in a reciprocal role, which was in agreement with the result of ALP activity and calcium deposition.

Gamma radiation also had a profound effect on the potential of adipogenic differentiation of BMSCs. Results showed that low dose of radiation, such as 2 Gy, increased the amount of adipocyte islands and TG on both surfaces. When it came to high dose of radiation, such as 4 Gy and 8 Gy, results were opposite, and the number of lipid droplets and TG significantly decreased. And it was suggested that higher than 4 Gy radiation had a serious inhibition on adipogenic differentiation [36]. However, as described by Chen et al., the dose of 9 Gy irradiation had no effect on adipogenic differentiation capability of MSCs [37]. No research so far reported that radiation could increase adipogenic differentiation in vitro. Some in vivo studies, nevertheless, found that radiation enhanced adipose tissue in bone marrow and bone marrows of the patients with radiation therapy were replaced by fat tissue partly [40]. On the basis of our study, it suggested that BMSCs had a tendency to differentiate into adipocyte under low dose of 2 Gy radiation.

Results also demonstrated that there were less adipose drops on SLA surface than that on PT surface. As adipogenic and osteogenic differentiation are reciprocal relationship, it was indicated that osteogenic differentiation of BMSCs was conducive on SLA surface [20, 21], which may explain why less adipose drops were observed on SLA surface. And the lipogenic related gene PPAR*γ*, translated to a critical nuclear receptor protein that it is required for adipocyte differentiation [41], was measured at the 7th day and the 14th day under different radiations. In the differentiation of MSCs, it also regulates the balance between osteogenic and adipogenic differentiation. As results suggested, the expression of PPAR*γ* was increased at 2 Gy radiation on both surfaces, but it dropped a lot after reaching 4 Gy radiation. Additionally, the level of PPAR*γ* was higher on PT surface than that on SLA surface, which indicated that smooth surface was better for adipogenic differentiation. However, it has been reported that low dose of radiation could reduce the expression of PPAR*γ* and then decrease the ability of both osteogenic and adipogenic differentiations [21].

Usually, researchers mainly study the effect of titanium plates or titanium sticks on osteogenesis of BMSCs; whether it has influences on adipogenesis is unknown. This was the first time that BMSCs were induced to adipogenic differentiation on Ti surface and lipid drops were directly seen on opaque materials through stereoscopic microscope. But how titanium surfaces affect the adipogenic differentiation of BMSCs and how radiation affects the BMSCs on PT surfaces or SLA surfaces are not clearly illustrated.

## 5. Conclusion

In this research, effect of different dose of *γ*-radiation on rat BMSCs on PT and SLA surfaces was investigated in vitro. The proliferation, spreading, and adhesive abilities of BMSCs were inhibited by *γ*-radiation in a dose-dependent manner. Radiation decreased the ability of osteogenic differentiation of BMSCs. In contrast, low dose of radiation promoted adipogenic differentiation of BMSCs on both surfaces. When the dose was higher than 4 Gy, however, adipogenic differentiation was suppressed. Additionally, on SLA surface, osteogenic differentiation was promoted no matter whether it was under radiation or not, and adipogenic differentiation is always suppressed. This suggests that rough surface may be better in improving the success rate of implant in radiation therapy patients than PT surface and also reveals the possible reasons accounting for high failure rate of implant in radiation therapy patients.

## Figures and Tables

**Table 1:** The sequence of the primers used for gene expression analysis.

Gene	Forward primer (5′-3′)	Reverse primer (5′-3′)
Runx2	AGTAAGAAGAGCCAGGCAGGTG	GTGTAAGTGAAGGTGGCTGGATAG
OCN	ACCCTCTCTCTGCTCACTCTGC	TTCACCACCTTACTGCCCTCC
PPAR*γ*	CCAGGCTTGCTGAACGTGAA	TGGAGCACCTTGGCGAACA
P53	ACCATCATCACGCTGGAAGACT	CTGGTGGGCAGTGCTCTCTT
GAPDH	TATGACTCTACCCACGGCAAGT	ATACTCAGCACCAGCATCACC

Runx2: runt-related transcription factor 2, OCN: osteocalcin, PPAR*γ*: peroxisome proliferator-activated receptor gamma, and GAPHD: glyceraldehyde-3-phosphate dehydrogenase.
